# Editorial: Linguistic biomarkers of neurological, cognitive, and psychiatric disorders: verification, analytical validation, clinical validation, and machine learning

**DOI:** 10.3389/fpsyg.2024.1454225

**Published:** 2024-07-26

**Authors:** Ratree Wayland, Kevin Tang, Si Chen

**Affiliations:** ^1^Department of Linguistics, College of Liberal Arts and Sciences, University of Florida, Gainesville, FL, United States; ^2^Department of English Language and Linguistics, Institute of English and American Studies, Faculty of Arts and Humanities, Heinrich-Heine-Universität Düsseldorf, Düsseldorf, Germany; ^3^Department of Chinese and Bilingual Studies, Faculty of Humanities, Hong Kong Polytechnic University, Kowloon, Hong Kong SAR, China

**Keywords:** neurodegenerative disease, autism, cognitive impairments, dementia, mental health stuttering, concussion, respiratory health

## Introduction

Speech production is a complex process involving the coordination of over 100 muscles across the respiratory, articulatory, and phonation systems. This intricate coordination makes speech a valuable source of biomarkers for various diseases. By analyzing speech production, we can gain insights into neuromuscular and psychological conditions, making it a powerful tool for the early detection and monitoring of these disorders as evidenced by the diverse studies in this Research Topic. These studies leverage and develop innovative methodologies to uncover the diagnostic potential of speech characteristics.

**Figure 1:**
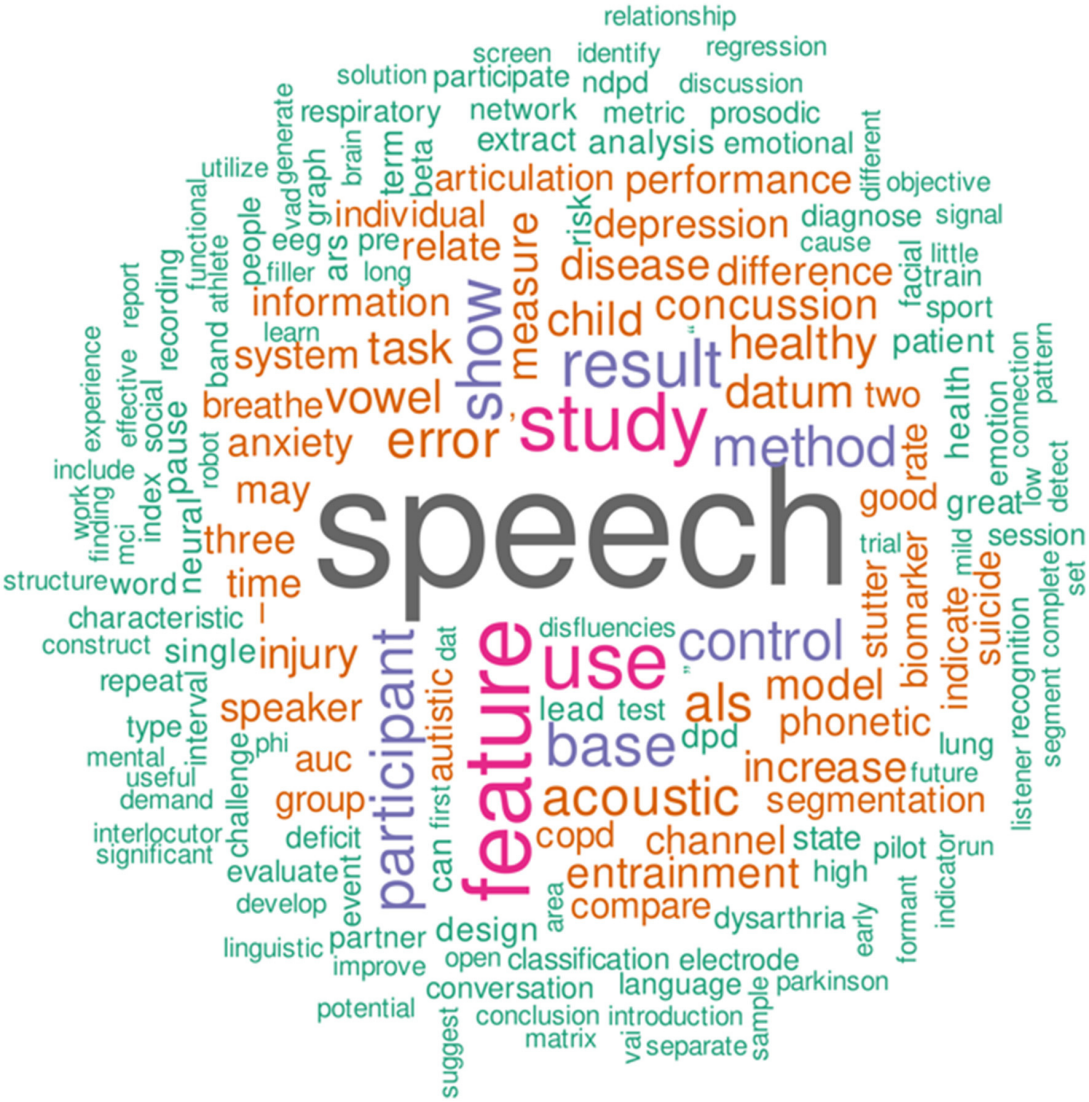
A word cloud which represents the 200 most frequent words found in the abstracts of the articles included in this special issue. The words were converted to lower case, English stop words were removed, and lemmatization was performed using the R libraries “tm” (Feinerer et al., 2008; Feinerer and Hornik, 2024) and “textstem” (Rinker, 2018). The resulting lemmas were visualized using the R library “wordcloud” (Fellows, 2018).

Nine Original Research articles were accepted in this Research Topic out of 17 submissions. With each paper having on average 5.2 authors, the interdisciplinary nature of this Research Topic is apparent. The nine articles cover seven broad types of disorders, including neurodegenerative diseases (Dash et al.; Roland et al.), neurodevelopmental disorders (Hong et al.), cognitive impairments (Oh et al.), psychological and emotional disorders (Cohen et al.; Chao et al.), respiratory health (Zeng et al.), concussion (Patel et al.) and stuttering (Barrett et al.). To illustrate what the nine articles cover, a word cloud (Figure 1) was generated showing the 200 most frequent words found in the abstracts of the articles.

## Neurodegenerative diseases

Dash et al. explore the use of magnetoencephalography (MEG) to identify neural biomarkers for Amyotrophic Lateral Sclerosis (ALS). By analyzing neuromagnetic patterns during speech tasks, their study identifies distinct beta band activity as a potential diagnostic marker, achieving high accuracy in single-trial classifications. Roland et al. focus on detecting early speech biomarkers of dysarthria in Parkinson's disease (PD) through vowel articulation analysis. Their use of vowel triangle areas (tVSA) and vowel articulation index (VAI) effectively distinguishes between dysarthric and non-dysarthric PD patients, highlighting the potential of speech analysis for early detection and differentiation in neurodegenerative diseases. These studies underscore the potential of advanced neural and acoustic analyses in identifying early, subtle markers of neurodegeneration.

## Neurodevelopmental disorders

Hong et al. demonstrate that phonetic entrainment, where people adjust their speech to match their partner's phonetic features, is challenging for individuals with Autism Spectrum Disorder (ASD). Using a social robot to control speech variability during conversations, the study found autistic children matched their typically developing (TD) peers in vowel formants and mean fundamental frequency (f0) but struggled with f0 range entrainment. This highlights the potential of human-robot interactions for assessing phonetic entrainment in autistic children.

## Cognitive impairments and dementia

Oh et al. focused on the differentiation of cognitive impairments and various forms of dementia through speech analysis. They investigate whether prosodic features can distinguish between Alzheimer's type dementia (DAT), vascular dementia (VaD), mild cognitive impairment (MCI), and healthy cognition. By identifying key features such as pitch, amplitude, rate, and syllable, they demonstrate the feasibility of using acoustic measures as diagnostic tools for cognitive conditions. This approach is complemented by listener perceptions of emotional prosody, which further validate the acoustic findings. These insights into speech characteristics offer a non-invasive and potentially scalable method for early diagnosis and differentiation of cognitive impairments.

## Psychological and emotional disorders

Speech analysis also extends its utility to the realm of psychological and emotional disorders. Cohen et al. evaluate a multimodal dialog system (MDS) for characterizing mental states in individuals with depression, anxiety, and suicide risk. By integrating speech, language, and facial movement biomarkers, their system offers a comprehensive approach to remote patient monitoring. The ability to analyze multimodal data not only improves classification performance but also provides a scalable solution for ongoing mental health assessment. Chao et al. introduce a novel ResGAT emotion recognition framework, which combines residual networks and graph attention networks, to enhance emotion recognition from EEG data. This method effectively captures spatial and connection information, significantly improving the accuracy of emotion recognition. These studies highlight the potential of speech and multimodal analysis in identifying and monitoring psychological and emotional states, paving the way for more effective mental health interventions.

## Speech and respiratory health

The link between speech and respiratory health is another critical area of exploration. Zeng et al. investigate how speech breathing can be linked to lung function in chronic respiratory diseases. Their study uses articulation tasks to challenge and quantify speech articulation and breathlessness. The increase in pause ratios over successive runs provides quantifiable evidence of respiratory demand, suggesting that speech tasks can effectively assess respiratory health. This approach offers a non-invasive method for monitoring chronic respiratory conditions, potentially leading to better disease management.

## Speech and concussions

Speech analysis also shows potential in assessing neurological impacts from mild head injuries. Patel et al. analyze speech error rates in athletes post-concussion, revealing significant increases in pauses and time fillers. This study demonstrates that even mild head injuries can result in detectable speech changes, suggesting that speech analysis could serve as a diagnostic tool for concussions. The ability to identify subtle speech errors provides an additional layer of assessment for sports-related injuries, contributing to more comprehensive care for athletes.

## Speech disorders

Finally, the application of speech analysis to detect and manage speech disorders is exemplified by Barrett et al.'s study on automatic recognition of stutters (ARS). By comparing event-based and interval-based segmentation methods, their research shows that event-based segmentation more effectively preserves stutter boundaries and types, leading to better ARS performance. This study emphasizes the importance of segmentation techniques in speech analysis and suggests that refined methods and larger datasets could further improve ARS systems. The findings point to the potential of automated speech analysis in supporting interventions for speech disorders, enhancing the ability to monitor and manage conditions like stuttering.

## Conclusion

The studies presented in this Research Topic illustrate the potential of speech analysis as biomarkers for a range of neuromuscular and psychological disorders. The innovative methodologies and findings underscore the importance of further research in this field. By leveraging advanced acoustic, neural, and multimodal analyses, as well as machine learning and automatic speech recognition algorithms, researchers can enhance diagnostic accuracy and patient care, paving the way for early intervention and personalized treatment strategies. The preliminary nature of the findings of some studies calls for more research involving larger subject groups and patient populations with various diseases to validate the differential power of speech-based biomarkers across different conditions.

## Author contributions

RW: Writing – review & editing, Writing – original draft. KT: Writing – review & editing, Writing – original draft, Visualization. SC: Writing – review & editing, Writing – original draft.

## References

[B1] Feinerer I. Hornik K. (2024). tm: Text Mining Package. R package version 0.7-13. Available online at: https://CRAN.R-project.org/package=tm

[B2] Feinerer I. Hornik K. Meyer D. (2008). Text mining infrastructure in R. J. Stat. Softw. 25, 1–54. 10.18637/jss.v025.i05

[B3] Fellows I. (2018). wordcloud: Word Clouds. R package version 2.6. Available online at: https://CRAN.R-project.org/package=wordcloud

[B4] Rinker T. W. (2018). textstem: Tools for Stemming and Lemmatizing Text Version 0.1.4. Buffalo, NY. Available online at: http://github.com/trinker/textstem

